# Effect of Apple Cider Vinegar Intake on Body Composition in Humans with Type 2 Diabetes and/or Overweight: A Systematic Review and Meta-Analysis of Randomized Controlled Trials

**DOI:** 10.3390/nu17183000

**Published:** 2025-09-19

**Authors:** Alberto Castagna, Yvelise Ferro, Francesca Rita Noto, Rossella Bruno, Analucia Aragao Guimaraes, Carmelo Pujia, Elisa Mazza, Samantha Maurotti, Tiziana Montalcini, Arturo Pujia

**Affiliations:** 1 Department of Medical and Surgical Sciences, University “Magna Græcia”, 88100 Catanzaro, Italy; a.castagna@unicz.it (A.C.); yferro@unicz.it (Y.F.); francescarita.noto@unicz.it (F.R.N.); rossellabruno7@gmail.com (R.B.); pujia@unicz.it (A.P.); 2 O.U. Clinical Nutrition, “Renato Dulbecco” Hospital, 88100 Catanzaro, Italy; 3 Department of Clinical and Experimental Medicine, University “Magna Græcia”, 88100 Catanzaro, Italy; elisamazza@unicz.it (E.M.); smaurotti@unicz.it (S.M.); 4 Research Center for the Prevention and Treatment of Metabolic Diseases, University “Magna Græcia”, 88100 Catanzaro, Italy

**Keywords:** apple cider vinegar, flavonoids, weight management, body composition, obesity, meta-analysis

## Abstract

**Background:** Apple cider vinegar (ACV) is a naturally fermented beverage with potential metabolic health benefits; however, its effects on weight loss remain controversial. This systematic review and meta-analysis of randomized controlled trials (RCTs) was conducted to assess the effect of ACV on anthropometric measurements in adults. **Methods:** We performed a systematic search of PubMed, Web of Science, Scopus, and CENTRAL up to March 2025 for randomized controlled trials (RCTs) in adults (≥18 years) evaluating the effects of ACV for ≥4 weeks on body composition parameters. Primary outcomes included changes in body weight, BMI, waist circumference, and other anthropometric measures. Risk of bias was assessed using the Revised Cochrane Risk-of-bias tool. **Results:** Out of 2961 reports screened, 10 RCTs comprising a total of 789 participants were eligible for inclusion in this meta-analysis. The pooled results using a random-effects model showed that daily ACV intake significantly reduced body weight [SMD: −0.39; 95% CI: −0.63, −0.15; *p* = 0.001; *I*^2^ = 62%], BMI [SMD: −0.65; 95% CI: −1.05, −0.26; *p* = 0.001; *I*^2^ = 83%], and WC [SMD: −0.34; 95% CI: −0.67, −0.02; *p* = 0.04; *I*^2^ = 61%]. However, no significant effects of ACV were observed on the other body composition parameters analyzed. Sensitivity analyses excluding high-risk-of-bias studies confirmed the robustness of ACV’s beneficial effects on body weight and BMI. Subgroup analyses suggested that ACV consumption significantly improved anthropometric parameters when administered for up to 12 weeks, at a dose of 30 mL/day, and in adults who were overweight, obese, or had type 2 diabetes. **Conclusions:** Overall, this meta-analysis suggests that ACV supplementation may be a promising and accessible adjunctive strategy for short-term weight management in adults with excess body weight or metabolic complications.

## 1. Introduction

Overweight, obesity, and type 2 diabetes mellitus (T2DM) represent a global health emergency with significant economic repercussions for health systems worldwide [1,2,3,4,5]. In 2021–2022, it was estimated that over one billion individuals were affected by obesity globally [5,6], and 508 million people were living with T2DM [5].

Moreover, the continued increase in overweight and obesity among children and adolescents worldwide is expected to lead to a future increase in obesity rates among the adult population as well [7], with current estimates suggesting that up to four billion people, or over 51% of the world’s adult population, could be affected by 2035 [3]. Since obesity is a major risk factor for T2DM, rising obesity rates are expected to drive a corresponding increase in T2DM prevalence globally [5].

In addition, obesity is also a major risk factor for non-communicable diseases (NCDs), such as cardiovascular diseases, and several types of cancer [8]. Among these, cardiovascular mortality and tumors, linked to obesity, will have an ever-increasing relevance in the coming years [9,10].

There is therefore an urgent need to implement effective interventions to prevent and counteract overweight, obesity, and related complications.

Several effective weight loss strategies are currently available, including lifestyle changes (e.g., reduced caloric intake and increased physical activity), medications, medical devices, and surgical interventions [11,12]. However, long-term weight maintenance remains highly challenging, and weight regain is common [13,14,15].

Furthermore, although obesity pharmacotherapy and metabolic and bariatric surgery can be effective treatments, they can also lead to significant and, in some cases, serious side effects [16,17,18,19]. For example, anti-obesity drugs can cause adverse effects such as nausea, diarrhea, headache, or, in rarer cases, cardiovascular complications [16,17,18]. Furthermore, not all individuals with obesity are eligible for or have access to these therapeutic interventions, which may be limited by medical contraindications, geographic barriers, or economic constraints [20,21,22]. Therefore, alternative approaches are increasingly being considered. Recently, there has been growing interest in using various plant parts—including roots, stems, leaves, flowers, seeds, and fruits—or their derivatives, which are rich in polyphenols, for the prevention and management of obesity and its metabolic complications [23,24,25].

In particular, apple cider vinegar (ACV) is one of the most commonly used plant-based products globally, both as a preservative and as a food flavoring [26]. ACV is produced through a two-step fermentation process of apple juice: in the first step, yeasts and bacteria convert fruit sugars into ethanol, while in the second step, ethanol is oxidized into acetic acid [26]. In addition to acetic acid, which is the main active ingredient, ACV contains numerous bioactive molecules, including polyphenolic compounds such as caffeic acid, catechin, gallic acid, and ferulic acid [26,27], which may exert beneficial effects on human health. In particular, ACV has shown promising effects in improving glycemic control and insulin sensitivity [28,29,30], lipid profiles [28,29,30], and reducing oxidative stress [31], making it a potential adjunctive strategy in the management of metabolic disorders such as obesity, diabetes, and dyslipidemia [28,29,30,31]. Some studies suggest that ACV supplementation may reduce body weight and total fat mass [31,32,33].

However, despite the widespread consumption of ACV for its purported health benefits, scientific evidence supporting its anti-obesity effects remains limited. A recent meta-analysis [34], which examined studies conducted up to 2022, reported that ACV consumption had no significant effect on body mass index (BMI). However, this analysis did not consider body weight or other body composition parameters [34].

Thus, this review aims to examine the existing evidence on the effects of ACV consumption on weight management. Specifically, we conducted a meta-analysis of randomized controlled trials (RCTs) to evaluate the effect of ACV on body weight and body composition changes in adult individuals. The findings from this analysis may offer new insights into the role of plant-based products, such as ACV, in the prevention and management of overweight, obesity, and related complications.

## 2. Material and Methods

### 2.1. Selection and Search Strategy

This review was conducted following the Preferred Reporting Items for Systematic Reviews and Meta-Analyses (PRISMA) guidelines [35].

The protocol for the study was registered with International Prospective Register of Systematic Reviews (PROSPERO; CRD420251001293 registered on 4 March 2025) (https://www.crd.york.ac.uk/PROSPERO/view/CRD420251001293, accessed on 16 September 2025). The meta-analysis was conducted following the guidelines outlined in the Cochrane Handbook for Systematic Reviews of Interventions [36]. We performed a comprehensive search for relevant articles in four databases, including Web of Science, PubMed, Scopus, and the Cochrane Central Register of Controlled Trials (CENTRAL), without any time restrictions. Our search was focused on those studies in humans that explore the effect of ACV intake on body composition. The question considered in this review was “Are apple cider vinegar interventions effective in improving body composition in humans?”.

The search strategy included specific terms related to interventions (“acetic acid”, “vinegar”, “Apple cider vinegar”, “ACV”, and “fermented apple”) and body weight management (“obesity”, “weight”, and “BMI”, Supplementary Table S1). To ensure the selection of relevant and high-quality studies, the search terms also included keywords related to randomized clinical trials (“randomized”, “randomized”, “controlled clinical trial”) (Supplementary Table S1). The search strategies for PubMed were combined with filters dedicated to RCTs proposed by the Cochrane Collaboration. Furthermore, we also reviewed reference lists of relevant meta-analyses and reviews. The last search was performed from the inception of the electronic databases up until 27 March 2025.

### 2.2. Inclusion/Exclusion Criteria

This study was designed to evaluate the effectiveness of ACV for weight loss and changes in body composition. The review included studies that (1) involved adults aged ≥ 18 years; (2) included healthy individuals; or (3) included adults with obesity, metabolic syndrome, mild hypercholesterolemia, metabolic dysfunction-associated steatotic liver disease, prediabetes, diabetes, or hypertension. Only RCTs with a parallel or crossover design and an intervention duration of at least one month were included. We also included studies comparing apple cider vinegar, alone or combined with other nutritional interventions, to a placebo or another active control. Finally, we included studies reporting changes in body weight and/or other outcomes, such as body mass index (BMI), waist circumference (WC), waist-to-hip ratio (WHR), and fat mass (FM), either comparing intervention and control groups during the study period or providing sufficient information to calculate these values. The exclusion criteria were as follows: (1) in vitro or animal studies; (2) RCTs involving children, adolescents, or pregnant or breastfeeding women; (3) studies in which ACV was not used as the intervention (i.e., other types of vinegar); and (4) studies without an active control group. Systematic reviews and meta-analyses performed to assess the effect of ACV intake on cardiometabolic risk factors were examined to identify additional RCTs not captured in the initial database search. All retrieved articles were examined, and titles and abstracts were independently screened reviewed by three authors (FRN, RB, and AAG) using the Rayyan application for systematic reviews to identify and eliminate duplicates, as well as assess eligibility based on the inclusion criteria. Initially excluded articles were reviewed by a fourth researcher (YF) to verify the decision. Any discrepancies between the first three researchers were resolved in consultation with this fourth author. Finally, the full texts of all selected articles were independently reviewed and discussed by the same researchers.

### 2.3. Data Extraction and Quality Assessment

Data from each article were collected in an Excel spreadsheet by two independent authors (YF and FRN). We created a standardized form to extract the following data: (1) first author’s surname; (2) year of publication; (3) study location; (4) study design (parallel or crossover); (5) number of participants in each group; (6) participants’ health status; (7) mean age and gender; and (8) dosages, types, and duration of the intervention and control. Moreover, we extracted post-intervention changes in weight, BMI, WC, WHR, and FM, reported as mean (standard deviation; SD), mean (standard error; SE), or mean difference with 95% confidence intervals (CI). Any missing or unclear data were obtained via email communication with the corresponding authors.

The methodological quality of the RCTs included in this meta-analysis was assessed using the Revised Cochrane Risk-of-Bias Tool for Randomized Trials (RoB 2). Two authors (YF and RB) independently conducted the risk-of-bias assessment using standardized data extraction forms. Any disagreements were resolved through discussion with a third reviewer (AC) until consensus was reached. We evaluated the following domains: random sequence generation, allocation concealment, blinding of participants and personnel, blinding of outcome assessment, management of missing data, and completeness of outcome reporting [37]. Each item was categorized as having low, unclear, or high risk of bias. Domains with methodological flaws potentially affecting the results were classified as high-risk, whereas those without such flaws were classified as low-risk. When information was insufficient for a definitive judgment, the domain was considered to have an uncertain risk. When there was insufficient information to make a definitive judgment, the domain was classified as having “uncertain risk.” Studies in which all domains were rated as low-risk were considered high-quality, indicating minimal risk of bias.

### 2.4. Statistical Analysis

The meta-analysis included all studies that reported relevant outcomes, provided data for the endpoints assessed in this review, and included homogeneous interventions. The primary outcome was change in body weight (kg). Values reported in pounds were converted to kilograms. Secondary outcomes included changes in BMI and WC, as well as, when applicable, changes in WHR and FM. To calculate the overall effect size, standardized mean differences (SMDs) and standard deviations (SDs) were extracted for all body composition outcomes. Outcomes reported as standard errors (SEs) or confidence intervals (CIs) were converted to SDs [35]. A random-effects model was applied for all analyses. We performed subgroup analyses based on study duration (4–8 weeks vs. 12 weeks), ACV dosage (5–15 mL/day vs. 30 mL/day), and type of control (water vs. other comparators). Additionally, analyses were stratified by participant characteristics, specifically comparing overweight/obese individuals and those with type 2 diabetes (T2DM). Heterogeneity between studies was estimated using the chi-square test and quantified using the *I*^2^ inconsistency measure, where 25%, 50%, and 75% indicate low, moderate, and high heterogeneity, respectively [38]. Values above 50% indicate substantial heterogeneity [38]. For all outcomes, sensitivity analyses were conducted by removing studies assessed as having a high risk of bias. We conducted a random-effects meta-regression using Stata version 16 to explore how certain moderators—total number of patients, number of administrations, year of publication, baseline BMI, study duration, and ACV dosage—could account for the observed heterogeneity in effect sizes. The selection of moderators was defined a priori. Heterogeneity across studies was assessed using *I*^2^, Tau^2^, and Cochran’s Q test (chi-square). Finally, funnel plots were generated to visually evaluate study precision and potential publication bias. All other statistical analyses were performed using the Review Manager Software (version 5.4; Cochrane Training).

## 3. Results

**Figure 1:**
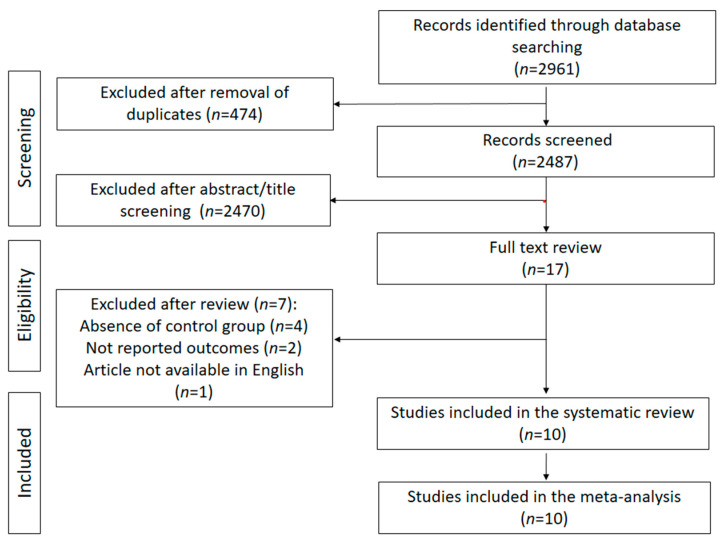
PRISMA flow-chart of the research strategy.

A total of 2961 papers were identified for this study [162 from PubMed, 1141 from Scopus, 166 from the Cochrane Library, and 1492 from Web of Science (Supplementary Table S1)]. After removing 474 duplicates, 2487 articles were screened for inclusion based on titles and abstracts (Figure 1). After 2470 papers were excluded, the 17 papers that remained underwent full-text assessment. A total of 7 papers were excluded (Figure 1), with 10 studies included in the meta-analysis [39,40,41,42,43,44,45,46,47,48] In particular, four studies were excluded from the selection because they did not include a control group, an essential requirement for the comparative evaluation of outcomes [49,50,51,52]. Finally, three studies were excluded: two for not reporting the outcomes of interest (weight, BMI, WC, WHR, and FM) [53,54] and one because it was published in a language other than English [55].

The main characteristics of the included studies and the types of interventions are reported in Table 1. A total of 10 studies, published between 2009 and 2024, were included in the analysis. The studies were mainly conducted in Asia (Iran, Pakistan, India, Japan) [39,40,41,42,43,45,46,47], in North Africa (Tunisia) [48], and in the Middle East (Lebanon) [44]. The number of subjects per study ranged from 26 to 126. The participants were adults with overweight, obesity, or type 2 diabetes, for a total of 861 subjects of both genders. The average BMI, when available, varied from 26.51 to 32.1 kg/m^2^. All studies included an intervention based on ACV, administered either in liquid form (5 to 30 mL per day) or as effervescent tablets [39], sometimes combined with recommendations for a healthy diet, a low-calorie diet, or exercise. The control groups received water, placebo with a similar taste, metformin, or only diet and exercise. The duration of the interventions varied from 4 to 12 weeks. The evaluated outcomes included anthropometric measurements and serum glucose and lipid concentrations.

In eight studies, a significant reduction in weight, BMI, or other body composition parameters was observed in the ACV group compared with controls. Among these, Abou-Khalil et al. [44], Khezri et al. [47], Roshan et al. [41], and Kannan et al. [39] reported a significant reduction in both in body weight and BMI and in metabolic parameters. In other studies, such as Kausar et al. [46], no significant differences in anthropometric measurements emerged, despite improvements in serum glucose and lipid profiles.

**Figure 2:**
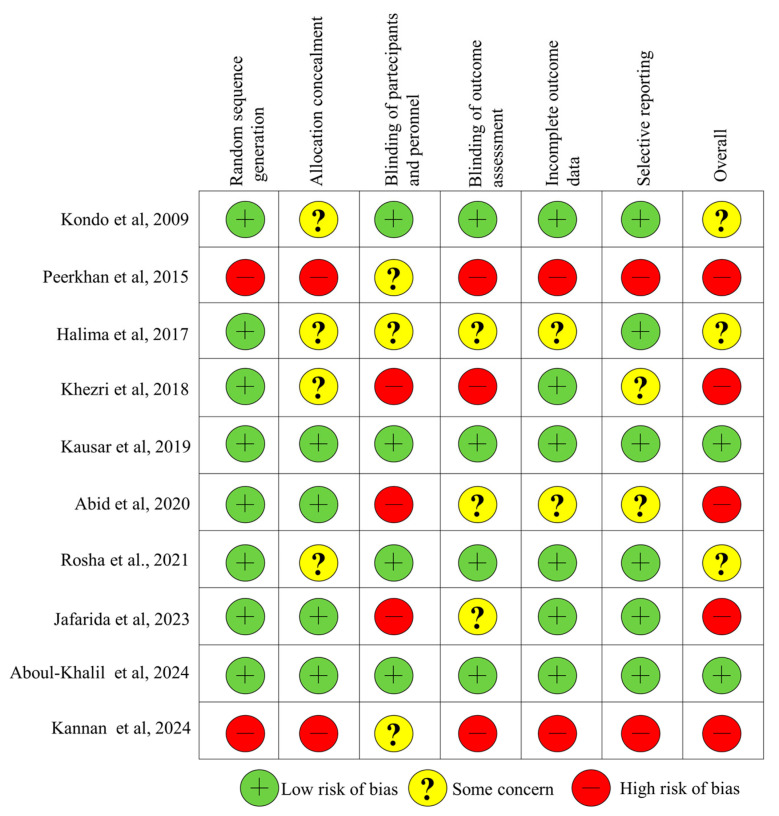
Assessment of the methodological quality of the included Studies [39,40,41,42,43,44,45,46,47,48].

Figure 2 presents the results of the Cochrane risk-of-bias tool used to assess the quality of the included studies. Of the ten studies, two were classified as having a low risk of bias across all evaluated domains [44,46]. Three trials showed an unclear risk of bias, specifically in relation to allocation concealment [40,41,48]. Finally, five studies were classified as having a high risk of bias in the domains of participant/personnel blinding or outcome assessment blinding [39,42,45,47].

**Figure 3:**
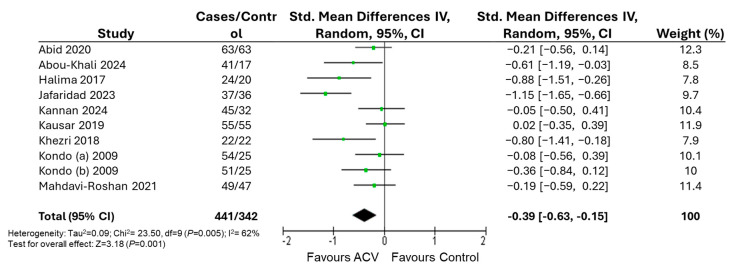
Forest plot of the effects of apple cider vinegar on body weight with intervention durations ranging from 4 to 12 weeks. Green dots represent the point estimates of each study, with horizontal lines indicating the corresponding 95% confidence intervals. The black diamond represents the overall effect estimate from the meta-analysis [39,40,41,42,44,45,46,47,48].

Figure 3 shows the meta-analysis results for body weight at 12 weeks. Nine studies provided data for quantitative analysis of body weight. The number of participants ranged from 22 to 63 in the intervention group and from 17 to 65 in the control group. A significant effect of ACV intake was observed for body weight (Figure 3). This corresponds to a mean difference (MD) of −7.45 kg in body weight after ACV intake. For body weight, meta-regression analyses showed that heterogeneity was significantly explained by both sample size (r = 0.66, *p* = 0.037) and number of administrations (r = 0.68, *p* = 0.031).

**Figure 4:**
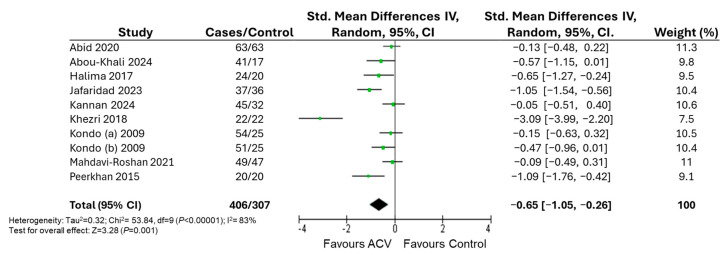
Forest plot of the effects of apple cider vinegar on BMI with intervention durations ranging from 4 to 12 weeks. Green dots represent the point estimates of each study, with horizontal lines indicating the corresponding 95% confidence intervals. The black diamond represents the overall effect estimate from the meta-analysis [39,40,41,42,43,44,45,47,48].

Figure 4 shows the results of the meta-analysis for BMI at 12 weeks. Nine studies reported data for quantitative analysis. The number of participants ranged from 20 to 63 in the intervention group and from 17 to 63 in the control group. A significant effect of daily ACV consumption was observed for BMI (Figure 4). This corresponds to an MD of −2.00 kg/m^2^ in BMI following ACV supplementation. For BMI, meta-regression analyses showed that heterogeneity no significant moderators were identified.

**Figure 5:**
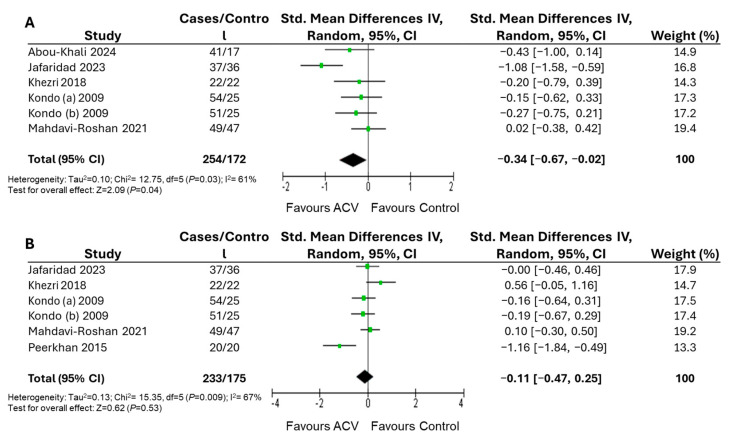
Forest plot of the effects of apple cider vinegar on waist circumference and waist-to-hip ratio with intervention durations ranging from 4 to 12 weeks. Green dots represent the point estimates of each study, with horizontal lines indicating the corresponding 95% confidence intervals. The black diamond represents the overall effect estimate from the meta-analysis [40,41,43,44,45,47].

Figure 5 shows the meta-analysis results for WC and WHR. For WC, there was a significant effect after ACV treatment (Figure 5A). This corresponds to an MD of −2.95 cm in WC following ACV treatment. For WC, meta-regression analyses showed that no significant heterogeneity moderators were identified. No significant effect of ACV intake was observed for WHR (Figure 5B).

The results of the sensitivity and subgroup analyses are reported in Table 2. Sensitivity analyses excluding studies at high risk of bias confirmed a significant effect of ACV on body weight and BMI, whereas no significant effects were observed for WC or WHR.

Regarding subject groups, significant reductions in body weight and BMI were observed both in overweight/obese individuals and in those with type 2 diabetes. However, for WC, only participants with type 2 diabetes experienced significant reductions, while effects were not significant in obese individuals (Table 2). In addition, significant effects of ACV intake on body weight, BMI, and WC were observed both in studies with intervention durations of 4–8 weeks and those lasting more than 9 weeks. Specifically, significant reductions in body weight were seen for both 4–8 weeks and 4–12 weeks, as well as for BMI in both shorter and longer durations. A significant effect on body weight was observed only at a dosage of 30 mL/day. Moreover, ACV treatment showed a dose-dependent effect on BMI. In particular, at doses of 5–15 mL/day, a SMD reduction of −0.24 [95% CI: −0.45, −0.03; *p* = 0.03] was observed, while a more pronounced reduction of −0.90 [95% CI: −1.53, −0.27; *p* = 0.005] was seen at 30 mL/day. No significant effect on WC was observed at any dosage (Table 2). Finally, no significant effects were detected for WHR across any subgroups.

Supplemental Figure S1 reports the funnel plots used for visual assessment of potential publication bias among the included studies.

## 4. Discussion

This review assessed the available evidence on the effectiveness of ACV consumption in the management of obesity. It provides an analysis of anthropometric parameters related to obesity in adult subjects. The main result of the meta-analysis clearly showed that supplementation with ACV is significantly effective in reducing body weight [SMD: −0.39; 95% CI: −0.63, −0.15; *p* = 0.001; *I*^2^ = 62%], BMI [SMD: −0.65; 95% CI: −1.05, −0.26; *p* = 0.001; *I*^2^ = 83%], and WC [SMD: −0.34; 95% CI: −0.67, −0.02; *p* = 0.04; *I*^2^ = 61%]. However, no significant effects were observed on WHR [SMD: −0.11; 95% CI: −0.47, 0.25; *p* = 0.53; *I*^2^ = 67%], nor was it possible to reliably evaluate changes in fat mass, as this outcome was reported in only one study [47].

The results of the sensitivity analyses conducted after excluding studies at high risk of bias strengthen the evidence supporting the beneficial effects of ACV on body weight and BMI. These findings indicate that the observed reductions are robust and not driven by low-quality trials. However, no significant effects were detected for WC or WHR, suggesting that the impact of ACV may be more evident on overall body mass rather than on fat distribution.

Subgroup analysis further confirmed the effectiveness of ACV in reducing body weight and BMI in both overweight/obese individuals and adults with type 2 diabetes (Table 2). However, significant reductions in WC were observed only in subjects with type 2 diabetes, while the reductions in overweight/obese individuals were not significant. Regarding the duration of the intervention, daily ACV intake significantly reduced body weight, as well as BMI, in both 4–8-week treatments and those lasting 12 weeks (Table 2). Finally, the dose of ACV proved to be a determining factor. A daily intake of 30 mL resulted in greater reductions in body weight and BMI compared to lower doses, ranging from 5 to 15 mL. Specifically, this dosage led to a significant reduction in body weight [SMD: −0.60; 95% CI: −1.05; −0.15; *p* = 0.009] and BMI [SMD: −0.90; 95% CI: −1.53; −0.27; *p* = 0.005] (Table 2).

These findings suggest that dietary supplementation with ACV may have a role in the prevention and treatment of obesity. Based on the estimated baseline body weight, the pooled effect size for weight reduction (SMD = −0.39) corresponds to an average weight loss of approximately 7.4 kg, or 9.4% of basal body weight. A weight loss in the range of 5% to 10% is well known to produce clinically significant improvements in cardiometabolic risk factors [56]; therefore, the observed effects of ACV intake may be not only statistically significant, but also clinically relevant.

However, as shown in Supplementary Figure S1, the funnel plots are not perfectly symmetrical, suggesting the potential presence of publication bias or small-study effects. In particular, the funnel plot for BMI reduction (Supplementary Figure S1B) appears notably asymmetrical, while those for body weight (Supplementary Figure S1A) and waist circumference (Supplementary Figure S1C) show slight asymmetry, indicating a possible but less pronounced systematic bias. In contrast, the funnel plot for WHR (Supplementary Figure S1D) includes a limited number of studies, making it difficult to draw reliable conclusions about potential bias. In addition, we performed meta-regression analyses to further explore potential sources of heterogeneity across studies. In particular, a significant effect was found for sample size and the number of administrations in relation to weight outcomes (*p* < 0.05). For BMI and WC, no significant moderators were identified. These findings suggest that study characteristics may partially contribute to heterogeneity. Therefore, the effect size of the interventions should be interpreted with caution. Thus, despite the observed funnel plot asymmetry and the results of the meta-regression, the robustness of our findings is supported by the consistent outcomes across sensitivity and subgroup analyses, as well as by the high methodological quality of most included studies.

The mechanisms by which ACV affects body weight and other anthropometric parameters are manifold. ACV contains many organic acids, including succinic, ascorbic, formic, citric, oxalic, and, in greater quantities, acetic acid, which is its major component [57]. Acetic acid has been shown to slow gastric emptying and increase the feeling of satiety, helping to reduce calorie intake [58]. The results show that the rate of gastric emptying depends on the stimulation of sensors to acids found in the proximal half of the small intestine. Acidity in the small intestine stimulates these sensors, causing the release of bicarbonates, which neutralize the acids and slow down gastric emptying [59].

In addition, recent studies on animal models have suggested that vinegar may act by improving β-cell function, thereby enhancing insulin secretion and reducing the postprandial glucose response while also limiting insulin spikes, which promote adipose tissue accumulation [60,61].

Furthermore, at the metabolic level, ACV can activate the enzyme AMP-activated protein kinase (AMPK). In particular, acetic acid, contained in ACV, is a key element in the synthesis of acetyl-coenzyme A (acetyl-CoA). During the synthesis of acetyl-CoA, adenosine triphosphate (ATP) is consumed and adenosine monophosphate (AMP) is produced, increasing the AMP/ATP ratio and activating the AMPK pathway. Activation of the AMPK pathway promotes glucose uptake and free fatty acid oxidation in skeletal muscle and inhibits gluconeogenesis, glycolysis, lipogenesis, and cholesterol formation in the liver [62,63,64].

Furthermore, ACV has been shown to have lipid-lowering effects, improving the lipid profile [30,47], thereby contributing to a reduction in visceral adiposity [47].

All these mechanisms support the role of ACV in reducing body weight, with more pronounced effects observed in individuals with T2DM. Furthermore, in our subgroup analysis, significant reductions in WC were observed exclusively among participants with T2DM, suggesting a more significant reduction in visceral fat in this subgroup. This finding is particularly relevant given the well-established association between abdominal adiposity and insulin resistance. Furthermore, two recent meta-analyses confirm the effect of ACV supplementation on glycemic endpoints in diabetic cohorts [28,34].

In patients with T2DM, in whom achieving therapeutic goals remains essential, ACV intake, in combination with lifestyle modifications and pharmacological strategies, could potentially improve anthropometric parameters, improve metabolic control, and reduce cardiovascular risk.

In addition to its metabolic effects, another potential mechanism involves the modulation of the gut microbiota. The polyphenols and other bioactive compounds present in ACV may favorably positively influence energy metabolism [65].

Finally, the results obtained in this meta-analysis regarding body composition are similar to those demonstrated in another meta-analysis on the effects of polyphenols derived from polyphenol-rich foods, supplements, or extracts in overweight and obese individuals [66]. These findings suggest that daily intake of ACV may represent a valuable aid in obesity management, offering a more affordable option with fewer side effects compared to pharmacological treatments for obesity [66].

### 4.1. Strengths and Limitations

This meta-analysis has several strengths. First, it includes only RCTs conducted on overweight and obese individuals or patients with type 2 diabetes mellitus, without the presence of other significant comorbidities. This helped reduce the influence of confounding clinical conditions, improving the consistency of the results and enhancing their applicability in clinical practice. Furthermore, all included studies had a randomized parallel-group design, ensuring high methodological quality and greater robustness of inferences. Subgroup analyses produced results consistent with those of the main analysis, confirming the effectiveness of daily ACV intake on anthropometric parameters.

However, despite these strengths, the meta-analysis also has some limitations. The main one concerns the high heterogeneity between the studies, probably due to differences in population characteristics, ACV dosages, administration methods, and types of comparators. Moreover, the different formulations of ACV used (liquid or effervescent tablets) may have influenced the observed efficacy, contributing to the variability in the results.

Furthermore, the duration of the interventions was relatively short, ranging from 4 to 12 weeks, which limits the ability to assess the long-term effects of ACV. Furthermore, it was not possible to conduct stratified analyses by gender or age group due to the lack of disaggregated data in the individual RCTs. Furthermore, subgroup analyses showed that the beneficial effect of ACV on body weight was significant only in studies without concomitant pharmacological treatments; therefore, it is possible to recommend ACV to these kinds of patients. Conversely, in trials including diet-only interventions (with or without exercise), the effect was not significant, but given the low number of studies, further research needs to be conducted to clarify the role of ACV in different clinical contexts. In addition, the inclusion of studies with heterogeneous control groups (water, placebo, diet, metformin) represents a further limitation, as it may compromise comparability and increase variability in the pooled results. Finally, the presence of funnel plot asymmetry, particularly for body weight and BMI outcomes, suggests a potential risk of publication bias, which should be considered when interpreting the magnitude of the observed effects.

### 4.2. Future Perspectives

To better understand the role of ACV in managing obesity and its related complications, long-term clinical trials with sufficiently large sample sizes are needed to allow stratified analyses based on demographic and clinical variables. Future RCTs should specifically focus on the effects of ACV on body weight and BMI as primary outcomes. Moreover, advanced and reliable methods for assessing body composition should be used to accurately measure the effects of ACV on fat mass, lean mass, and muscle mass, rather than relying solely on body weight. Finally, the use of standardized dosages and a uniform formulation as an intervention would help improve comparability between studies and the overall quality of the results.

## 5. Conclusions

This systematic review and meta-analysis highlights that ACV may be effective in the prevention and management of obesity in overweight or obese adults, as well as in individuals with type 2 diabetes. The results showed that daily intake of ACV was associated with a statistically significant reduction in body weight, BMI, and visceral adiposity. These findings were confirmed in sensitivity analyses excluding studies at high risk of bias, suggesting that the observed benefits are not solely due to lower-quality evidence.

In conclusion, ACV may represent a valuable nutritional intervention for managing obesity, particularly when combined with appropriate lifestyle modifications. However, further studies are needed to determine the true efficacy and safety of ACV in the prevention and treatment of obesity.

## Figures and Tables

**Table 1:** Characteristics of studies included in the meta-analysis.

Study, Year (Country)	Study Design and Duration (Weeks)	Type Participants (Number of Subjects)	BMI Mean ± SD (kg/m^2^)	Intervention	Comparator	Outcomes	Results
Abid et al., 2020 (Pakistan) [42]	Randomized, open-label, controlled trial (12 weeks)	Adults with newly diagnosed type 2 diabetes (*n*. 126)	30.56 ± 5.36	750 mg of metformin plus 2 tablespoons of ACV per day	750 mg of metformin alone per day	Weight and BMI, FBS, and HbA1c	Significant reduction only in FBS and HbA1c in the group that also took ACV compared to metformin alone
Abou-Khalil et al., 2024 (Lebanon) [44]	Four-arm, randomized, double-blind, placebo-controlled study (12 weeks)	Overweight and obese young adults (*n*. 57)	26.51 ± 3.5	5, 10, and 15 mL of ACV diluted in 250 mL of water daily	Water with taste and appearance similar to the treatment	Body composition, lipid and glucose parameters	The treatment group showed significant improvements in anthropometric outcomes compared to placebo
Halima et al., 2017 (Tunisia) [48]	Parallel, randomized, controlled clinical trial (4 weeks)	Adults diagnosed with type 2 diabetes (*n*. 26)	/	15 mL of ACV daily	Water	Body composition, lipid and glucose parameters	Significant reduction in BMI, FBS, and Tg in the treatment groups compared to placebo
Jafaridad et al., 2023 (Iran) [45]	Parallel, randomized, controlled, and open-label clinical trial (8 weeks)	Adults with type 2 diabetes (*n*. 80)	29.49 ± 5.09	30 mL of ACV diluted in 100 mL of water daily with recommendations for a healthy diet	Recommendations for a healthy diet	Body composition, lipid and glucose parameters	Significant post-treatment differences in body composition, HbA1c, and lipid profile were observed between the two groups
Kannan et al., 2024 (India) [39]	Parallel, randomized, controlled clinical trial (60 day)	Overweight/obese adults (*n*. 77)	/	Daily effervescent ACV tablet (~14 mL of ACV) combined with mild exercise and a low-sugar diet	Only the exercise and diet regimen	Body composition, lipid and glucose parameters. Food intake and various digestive issues	Significant reductions in anthropometric parameters and digestive issues were observed in the treatment groups compared to placebo
Kausar et al., 2019 (Pakistan) [46]	Single-blind, randomized, placebo-controlled trial (12 weeks)	Adults with type 2 diabetes (*n*. 110)	/	15 mL of ACV diluted in 200 mL of water daily, along with the usual diet and general dietary advice regarding high- and low-glycemic foods	15 ml of artificial flavor of apple cider vinegar, along with the usual diet and general dietary advice regarding high- and low-glycemic foods	HbA1c, FBS, and lipid and anthropometric parameters	Significant reductions in lipid and glycemic profiles were observed in the intervention group. No differences in anthropometric parameters between groups
Khezri et al., 2018 (Iran) [47]	Two-arm, parallel, randomized controlled trial (12 weeks)	Overweight/obese adults (*n*. 44)	32.1 ± 4.9	30 mL/day of ACV plus restricted-calorie diet	Restricted-calorie diet alone	Body composition and lipid parameters	Significant reduction in weight, BMI, LDL, and Tg and significant increase in HDL in the treatment group compared to the control group
Kondo et al., 2009 (Japan) [40]	Three-arm, parallel, randomized controlled trial (12 weeks)	Obese adults (*n*. 104)	27.2 ± 1.8	15 mL of ACV daily	Water	Body composition and lipid parameters	Significant reduction in BMI and Tg in the treatment group compared to the control group
Kondo et al., 2009 (Japan) [40]	Three-arm, parallel, randomized controlled trial (12 weeks)	Obese adults (*n*. 101)	27.0 ± 1.7	30 mL of ACV daily	Water	Body composition and lipid parameters	Significant reduction in BMI and Tg in the treatment group compared to the control group
Peerkhan et al., 2015 (India) [43]	Parallel, randomized, controlled clinical trial (12 weeks)	Adults with type 2 diabetes (*n*. 40)		30 mL of ACV daily	No intervention	Body composition, glucose and lipid parameters	Significant reduction in BMI, WHR, FBS, post prandial blood sugar levels, and HbA1c between groups
Roshan M et al., 2021 (Iran) [41]	Two-arm, randomized controlled trial (4 weeks)	Overweight/obese adults (*n*. 96)	29.91 ± 4.51	30 mL of ACV diluted in 250 cc of water daily	250 cc of water daily	Anthropometric measurements, lipid parameters, and FBS	Significant reduction in weight and total Chol in the treatment group compared to the control group

ACV: apple cider vinegar; Total Chol: total cholesterol; LDL: low-density lipoprotein; HDL: high density lipoprotein; Tg: triglyceride; FBS: fasting blood sugar; HbA1: glycosylated hemoglobin; BMI: body mass index; WHR: waist-to-hip ratio.

**Table 2:** Sensitivity and subgroup analyses of the effects of apple cider vinegar on each outcome.

Variables	Sensitivity Analysis		Number of Participants	Effect Size SMD (95% CI)	*I*^2^ (%)	*p*-Value
Body weight	Excluding studies at high risk of bias		463	−0.29 [−0.54, −0.04]	39	0.02
BMI	Excluding studies at high risk of bias		353	−0.33 [−0.55, −0.11]	0	0.004
WC	Excluding studies at high risk of bias		309	−0.17 [−0.40, 0.07]	0	0.16
WHR	Excluding studies at high risk of bias		251	−0.06 [−0.32, 0.20]	0	0.65
Variables	Subgroup		Number of participants	Effect Size SMD (95% CI)	*I*^2^ (%)	*p*-value
Body weight	Subject	Obese	430	−0.29 [−0.51, −0.08]	16	0.008
		T2DM	449	−0.44 [−0.85, −0.03]	78	0.03
	Duration	4–8 weeks	547	−0.32 [−0.61, −0.04]	58	0.03
		12 weeks	493	−0.27 [−0.50, −0.05]	31	0.02
	Dosage	5–15 mL/day	494	−0.24 [−0.48, 0.01]	41	0.06
		30 mL/day	289	−0.60 [−1.05, −0.15]	70	0.009
	Control	Water	353	−0.37 [−0.63, −0.11]	26	0.006
		Other	430	−0.41 [−0.83, 0.01]	78	0.06
		No drugs	657	−0.42 [−0.70, −0.15]	65	0.003
		Only diet with or without physical activity	304	−0.47 [−1.05, 0.10]	83	0.11
BMI	Subject	Obese	430	−0.65 [−1.24, −0.05]	88	0.03
		T2DM	283	−0.70 [−1.22, −0.18]	75	0.008
	Duration	4–8 weeks	470	−0.30 [−0.60, 0.00]	56	0.05
		12 weeks	365	−0.90 [−1.66, −0.15]	90	0.02
	Dosage	5–15 mL/day	384	−0.24 [−0.45, −0.03]	5	0.03
		30 mL/day	329	−0.90 [−1.53, −0.27]	90	0.005
	Control	Water	353	−0.33 [−0.55, −0.11]	0	0.004
		Other	360	−0.83 [−1.48, −0.19]	90	0.01
		No drugs	587	−0.73 [−1.17, −0.29]	84	0.001
		Only diet with or without physical activity	194	−1.35 [−2.76, 0.07]	95	0.06
WC	Subject	Obese	353	−0.17 [−0.39, 0.05]	0	0.12
		T2DM	73	−1.08 [−1.58, −0.59]	-	<0.001
	Duration	4–8 weeks	426	−0.25 [−0.56, 0.06]	53	0.11
		12 weeks	257	−0.25 [−0.51, 0.01]	0	0.06
	Dosage	5–15 mL/day	213	−0.26 [−0.63, 0.10]	0	0.16
		30 mL/day	213	−0.38 [−0.87, 0.11]	75	0.13
	Control	Water	251	−0.17 [−0.40, 0.07]	0	0.16
		Other	175	−0.66 [−1.52, 0.21]	80	0.14
		No drugs	426	−0.34 [−0.67, −0.02]	62	0.04
		Only diet with or without physical activity	117	−0.66 [−1.52, −0.21]	80	0.14
WHR	Subject	Obese	295	0.04 [−0.25, 0.34]	32	0.77
		T2DM	113	−0.55 [−1.69, 0.58]	87	0.34
	Duration	4–8 weeks	368	0.01 [−0.20, 0.22]	0	0.93
		12 weeks	239	−0.22 [−0.81, 0.37]	78	0.46
	Dosage	5–15 mL/day	79	−0.33 [−0.55, −0.11]	-	-
		30 mL/day	329	−0.11 [−0.55, 0.33]	74	0.63
	Control	Water	251	−0.06 [−0.32, 0.20]	0	0.65
		Other	157	−0.19 [−1.07, 0.70]	86	0.68
		No drugs	117	−0.11 [−0.47, 0.25]	67	0.53
		Only diet with or without physical activity	117	0.24 [−0.30, 0.78]	52	0.38

SMD: standardized mean difference; CI: confidence interval; BMI: body mass index; WHR: waist-to-hip ratio; Other: no intervention or a healthy diet, the usual diet with general dietary advice regarding high- and low-glycemic foods, caloric restriction, or metformin, with or without physical activity.

## Supplementary Materials

The following supporting information can be downloaded at https://www.mdpi.com/article/10.3390/nu17183000/s1, Figure S1: Funnel plot for changes in mean body weight, BMI, waist circumference, and waist-to-hip ratio; Table S1: The search strategy used for each database.
