# Supplementary material for: Effect of Apple Cider Vinegar Intake on Body Composition in Humans with Type 2 Diabetes and/or Overweight: A Systematic Review and Meta-Analysis of Randomized Controlled Trials
Source: Nutrients. 2025 Sep 19;17(18):3000. doi: 10.3390/nu17183000 (PMC12472926; doi:10.3390/nu17183000)
Supplement: Supplementary file 1 [file nutrients-17-03000-s001.zip › nutrients-3803108-supplementary.pdf]

Table S1. *The search strategy used for each database*

Databases.	Keywords	Results
PubMed	(("Acetic Acid"[MeSH Terms] OR "vinegar"[Title/Abstract] OR "acetic acid"[Title/Abstract] OR "Apple cider vinegar"[Title/Abstract] OR "ACV"[Title/Abstract] OR "fermented apple"[Title/Abstract]) AND ("randomized controlled trial"[Publication Type] OR "controlled clinical trial"[Publication Type] OR "controlled clinical trial"[All Fields] OR randomized[Title/Abstract] OR randomised[Title/Abstract] OR placebo[Title/Abstract] OR "clinical trials as topic"[MeSH Terms] OR "cross-over studies"[MeSH Terms] OR "cross-over studies"[All Fields] OR "cross over studies"[All Fields] OR "Cross-over study"[All Fields] OR "Cross over study"[All Fields] OR "clinical trial"[Publication Type] NOT animals[All Fields])) AND (Obesity OR weight OR BMI)	162
Scopus	(TITLE-ABS-KEY ((("vinegar") OR ("acetic acid") OR ("Apple cider vinegar") OR ("fermented apple"))) AND TITLE-ABS-KEY ((("randomized controlled trial") OR ("controlled clinical trial") OR ("randomized") OR ("randomised") OR ("placebo") OR ("clinical trials as topic") OR ("cross-over studies") OR ("cross over studies") OR ("Cross-over study") OR ("Cross over study") OR ("clinical trial")))) AND (("Obesity") OR ("BMI") OR ("Weight")) AND (LIMIT-TO (DOCTYPE,"ar")) AND (LIMIT-TO (LANGUAGE,"English"))	1141
Cochrane	(("vinegar") OR ("acetic acid") OR ("Apple cider vinegar") OR ("ACV") OR ("fermented apple")) AND (("randomized controlled trial") OR ("controlled clinical trial") OR ("randomized") OR ("randomised") OR ("placebo") OR ("clinical trials as topic") OR ("cross-over studies") OR ("cross over studies") OR ("Cross-over study") OR ("Cross over study") OR ("clinical trial")) AND ((“Obesity”) OR (“BMI”) OR ("Weight")) in Title Abstract Keyword - (Word variations have been searched)	166
Web of Science	(TS=((("vinegar") OR ("acetic acid") OR ("Apple cider vinegar") OR ("ACV") OR ("fermented apple")) AND (("randomized controlled trial") OR ("controlled clinical trial") OR (“randomized”) OR (“randomized”) OR (“placebo”) OR ("clinical trials as topic") OR ("cross-over studies") OR ("cross over studies") OR ("Cross-over study”) OR ("Cross over study") OR ("clinical trial") AND (("Obesity") OR("BMI") OR("Weight")))))	1492

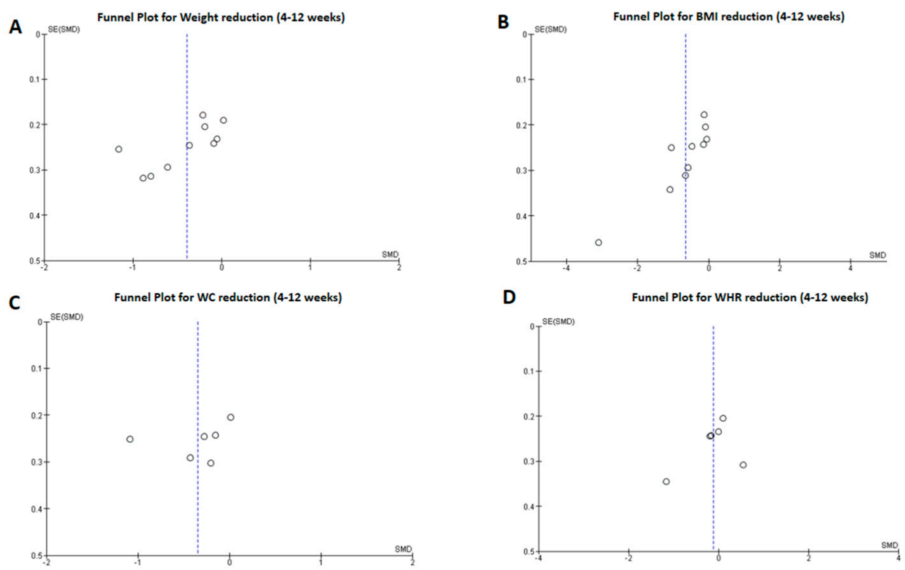

Figure S1. Funnel plot for changes in mean body weight, BMI, waist circumference and waist to hip ratio.
